# Transforming thyroid disease education: AI and virtual technologies in residency training

**DOI:** 10.3389/fendo.2026.1784933

**Published:** 2026-07-29

**Authors:** Shujian Xu, Cui Zhao, Nannan Sun, Qiang Gao

**Affiliations:** 1 Department of Thyroid Surgery, Binzhou Medical University Hospital, Binzhou, Shandong, China; 2 Department of Rehabilitation Medicine, Binzhou Medical University Hospital, Binzhou, Shandong, China; 3 Department of Operating Room, Binzhou Medical University Hospital, Binzhou, Shandong, China; 4 Second Affiliated Hospital of Soochow University, Suzhou, Jiangsu, China

**Keywords:** AI, medical education technology, residency training, surgical simulation, thyroid disease education, ultrasound training, virtual reality

## Abstract

Thyroid cancer is the most common endocrine malignancy, and standardized residency training is critical for cultivating competent thyroid specialists. However, traditional training faces limitations including insufficient standardized clinical exposure, patient safety concerns, and inconsistent skill assessment. This narrative review analyzes the applications, benefits, challenges, and future directions of artificial intelligence (AI) and virtual reality (VR) in thyroid disease-focused residency training. A literature search was conducted across PubMed, Scopus, and Web of Science, identifying 42 eligible English and Chinese studies published between 2015 and 2025. Results show that AI-driven systems enable objective, real-time assessment of thyroid ultrasound skills, enhance diagnostic decision-making for thyroid nodules, and support adaptive personalized learning. VR simulation platforms provide immersive, risk-free environments for repetitive practice of thyroid surgeries (e.g., thyroidectomy), with AI analytics further enabling precise skill evaluation and longitudinal progress tracking. Despite these advantages, significant obstacles persist: ethical and data security risks, technical limitations in anatomical fidelity and haptic feedback, professional acceptance and curricular integration issues, high economic costs, and potential weakening of humanistic competence. Future development should adhere to a “human-centered, technology-assisted” principle, focusing on core technological breakthroughs, standardized evaluation system construction, phased curriculum integration, and governance mechanism improvement. This review concludes that AI and VR are valuable adjuncts to traditional residency training, with the ultimate goal of cultivating thyroid specialists with both solid clinical skills and humanistic care.

## 1 Introduction

Thyroid cancer (TC) is the most common malignancy of the endocrine system. According to the data from the GLOBOCAN 2022 database, an estimated 821,214 new TC cases and 47,507 TC-related deaths occurred worldwide (1). The complex nature of TC demands physicians possess specialized skills in physical examination, ultrasonographic interpretation, fine-needle aspiration techniques, and surgical management—competencies that require extensive training and practice to develop. Residency training serves as a key component in developing high-level medical skills and is the main path to achieving sustainable development of health proficiencies and continuous improvement of medical standards of care (2). Since the standardized residency training system was established by the National Health and Family Planning Commission of the People’s Republic of China in 2014, the central and local governments, as well as hospital-based training centers, have invested substantial amounts in funds, human resource, and equipment for residency training programs. Traditional residency training programs often struggle to provide sufficient, standardized clinical exposure to thyroid diseases due to variability in patient presentations, limited training durations, and concerns about patient safety during the learning process (3).

The challenges in medical education are particularly pronounced in China, where a rapidly evolving healthcare system has identified significant gaps in physician training and patient outcomes (4). The 2009 launch of the China Thyroid Education Project highlighted deficiencies in standardized diagnosis and treatment, emphasizing the need for improved educational approaches. Similar challenges exist globally, prompting medical educators to explore innovative solutions to enhance training efficacy and standardization (5).

Recent technological advancements in artificial intelligence (AI) and virtual reality (VR) have created unprecedented opportunities to transform medical education (6). These technologies offer the potential to create immersive, high-fidelity learning environments that replicate complex clinical and surgical scenarios, enable deliberate and repetitive practice without any risk to patients, and provide objective, data-driven performance assessment (7). Specifically, AI algorithms can analyze complex patterns in surgical technique and diagnostic proficiency, offering residents personalized feedback, while VR simulations can replicate clinical scenarios with high fidelity (8). The integration of these technologies into residency programs represents a promising and necessary approach to addressing the longstanding educational challenges in thyroid disease management (9).It is essential to objectively and comprehensively discuss the current application status, achievements, obstacles, and challenges of these technologies in the resident training of thyroid specialists.

This comprehensive narrative literature review presents the current application status of AI and VR in the education of resident physicians on thyroid diseases, with a focus on the cultivation of diagnostic skills, surgical training, assessment methods, and implementation challenges. We searched PubMed, Scopus, and Web of Science using keywords such as “AI,” “virtual reality,” “thyroid disease,” “residency training,” and “surgical education.” The inclusion criteria were peer-reviewed articles published between 2015 and 2025, written in either English or Chinese, and focusing on the application of AI or VR in the training of resident physicians related to thyroid issues. The exclusion criteria include commentaries, editorials, and studies that are not in Chinese or English. We initially identified 87 studies, of which 42 met the inclusion criteria after full-text screening. Methodological quality assessment was performed using the Newcastle-Ottawa Scale for observational studies.

This review aims to: (1) describe current applications of AI and VR in thyroid-focused residency training; (2) summarize the results related to learning outcomes, skill acquisition, and competency assessment; (3) analyze the institutional, practical and ethical obstacles that exist during the implementation process; and (4) provide actionable recommendations for balanced, human-centered integration of technology into residency curricula. In conclusion, by integrating the research results from recent years and predicting the future exploration directions, we hope to provide scientific insights for educators and medical institutions, thereby strengthening the residency training programs through technological innovation.

## 2 AI-driven diagnostic skill development in thyroid disease

### 2.1 Intelligent ultrasound training systems

The integration of AI into ultrasound training has revolutionized how residents develop proficiency in thyroid imaging and assessment. Traditional ultrasound training requires extensive supervision and practice. Typically, a significant number of supervised scans are necessary to achieve basic proficiency, and there is a weak correlation between the number of scans and diagnostic accuracy (10). Recent technological innovations have addressed this challenge through the development of AI-powered assessment systems that provide objective performance metrics and real-time feedback (11).

A groundbreaking study published in *Medical Teacher* developed and validated a temporal AI model for automated micro-assessment of thyroid ultrasound skills in simulation-based training. Videos from 8 experts and 21 novices performing simulated thyroid ultrasound were analyzed using a Long Short-Term Memory (LSTM) networks with pre-trained ResNet-50 architectures for spatial feature extraction and long short-term memory layers to capture temporal movement patterns. Video sequences of different time segments were tested, with 50 seconds segments achieving the highest accuracy in distinguishing between expert and novice operators (70%) and F1 score (0.76). Bayesian updating and adaptive thresholding generated smoothed real-time competence probabilities. Experts demonstrated significantly longer durations above the expert-level threshold (15.71s vs. 9.31s, p=0.030) (12), reflecting sustained precise scanning rather than faster completion. In simple terms, this AI system observes the process of a trainee using a simulated ultrasound probe and a virtual patient’s neck to perform thyroid ultrasound scans. It assesses the trainee’s skills by observing how they move the probe, apply pressure, and obtain standard images. Previously, only experienced mentors could conduct the assessment through subjective methods. However, the AI can automatically and in real-time distinguish between experts and novices in simulated thyroid ultrasound training based on “the duration of expert-level stable operation”. Therefore, the educational value of these AI systems lies in their ability to provide nearly real-time and granular skill feedback for specific technical components (13). Residents can visualize their probe positioning, scanning trajectories, pressure application, and image optimization techniques, while simultaneously receiving a quantitative assessment of their proficiency. This approach transforms medical training from a subjective, mentor-guided process into an objective, data-driven learning experience, thereby accelerating the acquisition of skills, standardizing the assessment among residents, and addressing the resource constraints inherent in traditional expert evaluations (14).

### 2.2 AI-enhanced diagnostic decision making

Beyond technical skill development, AI systems are increasingly being deployed to enhance residents’ diagnostic decision-making capabilities (15). These systems integrate image analysis algorithms, and clinical data processing to support comprehensive assessment of thyroid nodules and cytology classification (16). During residency training, these tools serve as educational aids that reinforce pattern recognition and clinical reasoning skills (17).

Modern AI diagnostic assistants can analyze thyroid ultrasound images to automatically identify and characterize nodules, calculate cancer risk using standardized reporting systems like TI-RADS, and generate structured reports (18). When used in training contexts, these systems allow residents to compare their assessments with AI-generated interpretations, identifying discrepancies and refining their diagnostic approach (19). This iterative feedback process accelerates the development of diagnostic accuracy and clinical confidence (20).

Studies have demonstrated that AI system, designed as a supportive tool rather than a replacement, promises to revolutionize thyroid nodule diagnosis and management by providing a high level of diagnostic precision (20). The systems also facilitate personalized learning pathways by identifying specific diagnostic challenges individual residents face (e.g., distinguishing follicular lesions or interpreting cystic components) and providing targeted educational content and practice cases to address these areas of weakness (21).

### 2.3 Adaptive learning systems

AI technologies have enabled the development of adaptive learning platforms that customize thyroid disease education based on individual resident performance, learning pace, and specific knowledge gaps (6). These systems employ machine learning algorithms to analyze trainee interactions with educational content, assessment results, and simulation performance, subsequently modifying the curriculum to optimize learning efficiency (22). The comparison between AI-enhanced and traditional training methods in thyroid ultrasound education is detailed in **Table 1**.

**Table 1:** AI-enhanced vs traditional training methods in thyroid ultrasound education.

Feature	Traditional training	AI-enhanced training	Educational impact
Skill Assessment	Subjective evaluation by instructors	Quantitative micro-assessment of technique	Standardized competency measurement (12)
Feedback Timing	End-of-session or periodic	Real-time with every practice session	Immediate correction of technical errors (13)
Personalization	Limited adaptation to individual needs	Adaptive learning pathways based on performance	Addresses individual learning needs (21)
Proficiency Metrics	Time-based practice requirements	Objective quality thresholds	Ensures competence before clinical application (12)
Diagnostic Accuracy	Variable across institutions	Consistent improvement with AI guidance	Reduced practice variation (20)

The adaptive capabilities of these platforms are particularly valuable in thyroid disease education due to the multidisciplinary nature of the field, which encompasses endocrine physiology, surgical techniques, radiological interpretation, and pathological analysis (23). AI systems can integrate learning across these domains, identifying relationships between knowledge deficiencies in one area and performance limitations in another (24). For example, if a resident struggles with interpreting fine-needle aspiration cytology, the system might reinforce relevant anatomical, physiological, and pathological concepts (20).

These intelligent educational platforms also facilitate longitudinal assessment of competency development throughout residency training (25). By continuously tracking performance metrics across various diagnostic and procedural tasks, the systems can generate comprehensive competency profiles that help both residents and educators identify strengths, address weaknesses, and document milestones achievement (26). This data-driven approach to competency assessment provides objective evidence of readiness for independent practice (27).

## 3 VR and simulation in surgical training

### 3.1 Virtual simulation platforms

According to literature, the first decade of the twenty-first century was known as the “VR winter” with little public interest in this new technology. Since then, public interest in consumer-grade VR has increased, mostly for entertainment. Nevertheless, there was ongoing but limited research, mostly in corporate, academic, and military research laboratories around the world (28). In the fields of medicine and education, especially in medical education, the exploration of VR has never ceased. For instance, a randomized controlled trial showed that using VR self-conversations, based on motivational interviewing principles, may have benefits in helping people with obesity to enhance their readiness to change habits and self-efficacy, as well as reduce dysfunctional eating behaviors and anxiety (29). Another example is a research project conducted within the framework of the “Medical Training” program, which aims to develop an immersive VR learning platform based on AI. This platform utilizes intelligent, interactive virtual patients to simulate realistic clinical scenarios, enabling medical students to practice clinical decision-making. It is positioned to address numerous practical and ethical challenges in contemporary medical education, thereby better preparing students for future clinical practice (30). With the increasing interest and attention of the public to VR, the improvement of demand, the deep integration of interdisciplinary development, and the iterative update of technology, up to now, VR has become a transformative tool in the field of thyroid disease management surgery training, especially for complex thyroidectomy and fine needle aspiration biopsy (31).

The advent of digitalization has greatly changed the training methods in surgical education. Surgical simulators are increasingly being used to train a wide range of surgical skills, including suturing, knot tying, and laparoscopic surgery, and through the utilization of haptic technology, these simulators can generate highly realistic clinical environments, providing an innovative model for the acquisition of psychomotor skills (32). Popularly speaking, for example, in thyroid surgery education, modern VR thyroid simulators combine high-resolution 3D computer graphics with haptic (force-feedback) gloves or handheld controllers: trainees see a realistic 3D neck anatomy on a screen or head-mounted display, and when they “dissect” or “cut” with a virtual scalpel, the device resists like real tissue, simulating skin, fascia, thyroid gland, and even subtle differences between normal tissue and tumor. This haptic fidelity is particularly important for developing the delicate tissue manipulation skills required in thyroid procedures, where excessive traction can lead to laryngeal nerve injury or vascular complications (33).

Using the immersive VR platform, residents can repeatedly practice identifying anatomical landmarks, dissecting in precise tissue planes, and managing bleeding in a controlled environment that tolerates mistakes and allows for immediate corrective instruction (34). This benefits from the fact that current VR applications can display realistic visual effects, provide an immersive experience, and are reported to improve clinical decision making (35). This experience is rooted in two phenomena that M. Slater refers to “place illusion” and “plausibility illusion”. When both occur, they lead to realistic responses from VR participants (36). For example, “Surgical Theater Thyroidectomy VR Simulator” allows residents to gradually perform total/semi-thyroidectomy according to different anatomical structures and simulated bleeding crisis, including nerve identification and exposure, vascular ligation and hemostasis, and exploration and preservation of parathyroid glands (37). Each module presents progressively challenging scenarios that introduce anatomical variations, clinical pathological conditions, and intraoperative complications. AI-based predictive modeling, automated computer vision and image analytics, and robotic surgery are changing preoperative planning and intraoperative decision-making, with the ultimate aim of improving postoperative outcomes by reducing variability in surgery (38).

### 3.2 Comprehensive surgical skill development

VR simulation platforms facilitate the development of comprehensive skills in surgery (39). These skills not only cover basic surgical operation capabilities, but also include preoperative planning, intraoperative decision-making, and complication management - all of which are key components of surgical skills (40). Through repeated practice, residents develop the necessary psychomotor skills and cognitive frameworks required to cope with a variety of complex or rare scenarios (41). In traditional residency training, residents primarily observe or assist in practice, with little opportunity to independently lead complex cases or practice high-risk procedures. In contrast, VR allows trainees to practice the same complex scene operations dozens of times over and over again in a risk-free environment. This purposeful practice reinforces clinical decision making under pressure and fosters consistent judgment that cannot be reliably developed by standard bedside teaching alone. Research by Peterson et al. (42) has indicated that simulators can effectively address challenges specific to traditional health training programs, such as inconsistent exposure to rare cases and limited opportunities for deliberate practice. In addition, simulators also play a crucial role in introducing new teaching methods to teach complex medical content (43–45), enabling trainees to comprehensively grasp and integrate knowledge of different medical disciplines (46).

The ability to prevent and manage complications is a key indicator of surgical competence. Surgical simulation systems address this issue by incorporating a variety of potential complications, enabling trainees to develop and practice management strategies in a risk-free environment. For example, simulating total thyroidectomy for a large substernal goiter with tracheal compression and an abnormal recurrent laryngeal nerve -a rare but high-risk situation in clinical practice -allows residents to repeatedly practice preoperative planning, precise dissection around vital structures, and emergency airway management. Through repeated simulation exercises, patients can become familiar with the clinical manifestations of various complications and the corresponding treatment options, thus enhancing their ability and confidence to deal with emergencies in actual surgery (47). This higher degree of readiness not only reduces the risk of surgery, but also has the potential to promotes the improvement of postoperative recovery and quality of life of patients (48). Experiencing and dealing with rare but serious complications firsthand in a simulated environment helps residents better cope with practical challenges than traditional training methods that rely on casual clinical contact (47–49).

Although simulation systems have traditionally focused on improving individual surgical skills, their potential to facilitate team collaboration and communication in practical applications has been increasingly recognized. Studies have shown that the self-developed internet-based multi-user collaboration platform enables team members to participate in exercises that simulate surgical scenarios, thereby promoting effective communication and collaboration among team members (50). Unlike traditional operating room teamwork, which has a fixed division of roles and limited feedback mechanisms, multi-user VR allows multiple residents and faculty members to join a shared virtual operating room in real time, where they can act as chief surgeon, assistant, or observer. They communicated verbally and coordinated their actions, just as they would in a real operating room, and reviewed the process to achieve standardized, repetitive exercises in teamwork. The cultivation of such teamwork and communication skills is essential to improve the overall efficiency of the surgical team and enhance its ability to deal with complex clinical situations. By combining team training with technical skill development, these platforms can more comprehensively prepare residents for surgical practice than traditional isolated training approaches (51).

### 3.3 AI-enhanced performance analytics

The integration of AI-powered analytics into VR platforms has revolutionized surgical skill assessment in surgical training (52). The intelligent scoring system in surgical simulation platforms (general modular scoring system for evaluating proficiency levels of a surgery, GMSS) is designed to enhance procedural precision - a fundamental component of surgical competence- by providing meticulous operational assessment and immediate corrective feedback (53). These systems capture and analyze extensive performance data during simulated procedures, providing objective metrics on instrument handling, tissue manipulation efficiency, procedural flow, and error rates. For instance, the analysis platforms employ computer vision techniques to track surgical instruments and evaluate movement economy, path efficiency, and hand stability (54). Machine learning algorithms process this data to generate comprehensive performance evaluations that identify strengths and weaknesses with precision beyond human assessment capabilities (53). To address these weaknesses, the system enables targeted practice on specific technical deficiencies in key maneuvers such as cutting and suturing, incorporating continuous re-evaluation throughout the practice process. This targeted practice and detailed technical analysis provide residents with specific, actionable feedback to refine their surgical technique. This iterative process of rehearsal and assessment allows users to gradually develop the psychomotor skills and operational stability necessary for performing with greater confidence and accuracy in actual clinical procedures (55). Studies have shown that the VR-based thyroid surgical training platform can shorten the surgical procedure time by 5.5 minutes and increase the operation performance score by 1.5 points (56, 57).

Importantly, AI analytics are not meant to replace surgical mentors but to complement them. Senior surgical mentors are required to personally teach resident trainees the basic surgical procedures, provide evaluations based on their training performance, and share real surgical experiences. AI can track the fine operational details (such as the stability of the hand, the amount of force applied, the frequency of equipment collisions, etc.), which are often overlooked by human observers. It can operate outside the operating room or during non-working hours, providing consistent and unbiased feedback. Therefore, it does not replace imparting and guiding technological knowledge by experienced staff; instead, it quantifies progress so mentors can target their teaching more effectively.

The AI components also enable longitudinal progress tracking that documents skill development throughout residency training. By comparing current performance to previous attempts and established proficiency standards, the systems can objectively demonstrate competency acquisition and readiness for clinical practice (58). This data-driven approach to competency assessment helps program directors make informed decisions about advancement and credentialing while providing residents with clear evidence of their skill development (47). The key components, technical features, educational benefits, and assessment capabilities of the VR thyroid surgery simulation platform are detailed in **Table 2**.

**Table 2:** Key components of VR thyroid surgery simulation platforms.

Platform component	Technical features	Educational benefits	Assessment capabilities
Visual Simulation	High-fidelity 3D graphics, anatomical variability	Recognizes anatomical relationships	Identifies visual recognition errors (7)
Haptic Feedback	Tissue resistance simulation, instrument vibration	Develops appropriate tissue handling force	Measures force application accuracy (41)
Procedural Modules	Step-by-step procedure breakdown	Builds systematic surgical approach	Tracks protocol adherence (33, 47)
Complication Scenarios	Rare event simulation, emergency situations	Prepares for unexpected intraoperative events	Assesses crisis management skills (48)
Performance Analytics	Motion tracking, efficiency metrics, error detection	Provides objective feedback for improvement	Enables longitudinal progress monitoring (54)

## 4 Obstacles and challenges of AI and VR integration into medical education

Despite their promising potential in the field of medical education, these technologies still have limitations and disadvantages in the application of residency training due to the technical maturity, industry suitability and the humanistic nature of medical education, and their application faces obstacles and challenges.

### 4.1 Ethical risks and security challenges: data, equity and regulation

The implementation of AI is fraught with foundational concerns regarding data privacy, algorithmic bias, and equitable access. During the training process, the AI system needs a large amount of imaging data of thyroid surgery, such as ultrasound, CT and intraoperative video, which contains sensitive information of the patient. If it is not properly managed during storage or transmission, it may lead to serious privacy leakage. AI models, including large language models (LLMs), risk exposing sensitive patient information through adversarial attacks or data-sharing practices (59, 60). Meanwhile, data ownership is ambiguous and there is a lack of clear agreement between trainees and training institutions, leading to the risk of data abuse (61). For example, student performance data collected during VR training may be used for commercial purposes or improperly evaluated, over which the student lacks control (62). The standardized cases used in virtual training may not represent the diversity of real patients, such as patients with thyroid disease of different races, ages, and comorbidities. This data bias may lead to algorithmic bias in AI models, which in turn may affect residents’ mastery of surgical strategies for specific populations. In addition, the “black box” problem makes it difficult for residents to understand the reasoning process of AI and weakens the cultivation of critical thinking and independent judgment. At the same time, the “black box” nature of some AI algorithms creates challenges for understanding assessment methodology and ensuring fairness in evaluation (63).

At present, there is a lack of unified technical standards and certification systems for VR systems in the industry. Products from different manufacturers vary greatly in anatomical accuracy, haptic feedback quality, and AI evaluation logic, which makes it difficult to conduct horizontal comparison and standardized promotion of training effects, thus increasing the uncertainty of training results (64). Furthermore, the rapid pace of innovation has outstripped the development of coherent regulatory frameworks, leaving a critical governance gap for tools lacking formal approval (65).

### 4.2 Defects in simulation techniques: anatomy, haptic feedback, and dynamic factors

The existing VR system still has a certain degree of distortion in simulating the key anatomical details of thyroid surgery. The success of thyroid surgery is highly dependent on the exploration and exposure of the recurrent laryngeal nerve, the protection of the tiny blood supply of the parathyroid gland, and the anatomy of the dense adhesion of Berry ligament. However, most current VR models are constructed based on standard anatomical data, which makes it difficult to accurately reproduce these anatomical details that vary greatly from individual to individual. For example, common anatomical variations, such as non-recurrent variation of recurrent laryngeal nerve or ectopic parathyroid gland, are often ignored in standardized VR models, which leads to a certain degree of deviation between the anatomical cognition obtained by residents in simulation training and the real surgical scene, which weakens their ability to deal with complex cases (66).

In addition, the inadequacy of haptic feedback technology is another core drawback. The force feedback provided by VR devices is usually slightly stiff or has a short delay, which cannot truly simulate the elasticity, toughness, and cutting resistance of thyroid parenchyma, anterior cervical muscle group, and other tissues. A study on surgeons’ opinions regarding robotic-assisted surgery (RAS) and laparoscopic surgery (LS) found that the respondents generally regarded the tactile feedback defect of RAS as one of its major drawbacks (67). This distortion of tactile simulation directly affects the precise control of operation strength by residents, making it difficult for them to establish the same feeling and operating habits as the real operation in the simulation (68).

The current AI-VR system has shortcomings in simulating the dynamic changes during thyroid surgery. In real surgery, bleeding, respiratory movement, carotid artery pulse and other dynamic factors have a certain impact on the operation field and operation process. However, most simulation systems use static or preset scenarios, which cannot simulate intraoperative emergencies in real time, such as continuous bleeding of thyroid stump. Finally, the simulated environment generally lacks a realistic reduction of operative time pressure. Residents are prone to form “slow and steady” operation habits in virtual training without time limit, which is contrary to the time pressure of rapid hemostasis and consecutive operations in real surgery, and is not conducive to cultivating their ability to operate efficiently and accurately under time pressure (69).

### 4.3 Implementation barriers: professional acceptance and curricular integration

In addition to the technical obstacles, practical application also faces problems such as professional acceptance and curricular integration. Many educators are still cautious about the integration of AI and VR into the medical education system, questioning their actual teaching effect and significance, as well as their comprehensive evaluation ability (70, 71). Some people believe that the skills learned by residents in the VR environment are often difficult to transfer directly to real surgery or medical treatment scenarios. The fundamental reason is that the simulation environment lacks the uncertainty of real patients, time pressure, team cooperation and other key elements (72). Failure of skill transfer not only wastes valuable training resources, but also may lead to clinical errors due to false confidence generated by trainees, which may affect patient safety (73). Some people even argue that in thyroid surgery, a field that highly relies on fine anatomy and tactile judgment, the transmission of knowledge about soft tissue layers and tension control in apprentice transmission is difficult to be completely replaced by quantitative data (74).

The effective integration of AI and VR technology into the existing standardized residency training curriculum system faces great challenges in curriculum integration, and it will also cause profound reflection on the role orientation of teachers. First of all, it costs a lot of time and manpower to reallocate courses and train teachers to master the new teaching tools in the process of teaching integration. Secondly, it is a long adaptation process for teachers to redefine their role, recognize the auxiliary role of new teaching tools, and avoid excessive dependence on AI and VR system. In addition, it is difficult to integrate the AI platform with the hospital’s existing information systems (such as electronic medical records and image archiving systems), leading to the phenomenon of data siloing. The difficulty of real-time and cross-scene data integration is also a realistic obstacle to curriculum integration.

### 4.4 Economic burden and resource allocation limitations: costs, returns, and accessibility

The initial deployment and continuous maintenance costs of high-end VR surgical simulators and AI systems are extremely high, which poses a heavy economic burden to most teaching hospitals. For instance, the price of high-end simulators such as the Da Vinci Surgical System can range from several hundred thousand to several million dollars. In addition, the cost of system maintenance and upgrade is also high, including continuous software updates, hardware calibration and technical support (75). The training of surgeons and hospital engineers also constitutes a significant hidden cost.

Although AI and VR technologies have the potential to shorten the training cycle, their return on investment is difficult to quantify. For example, the existing research conclusions on the training effect are not completely consistent. Some studies have shown that simulation training can reduce the operation time and improve the proficiency of the operation (76), but other studies have pointed out that these techniques do not significantly reduce the incidence of complications or improve the passing rate of the students (77). The causal relationship between the improvement of training effect (such as the improvement of trainees’ ability) and the improvement of patient prognosis is complex and interfered by many factors, making it difficult to accurately calculate the return on investment (73). High costs may lead to waste of resources if they cannot be translated into measurable clinical benefits.

Large teaching hospitals in developed areas take the lead in deploying AI and VR systems by virtue of their economic and resource advantages, while medical institutions in resource-poor, poor or remote areas can only rely on traditional training models, which leads to a widening gap in the form and content of resident training (73). This technology gap may further exacerbate the uneven distribution of medical personnel (78).

### 4.5 Humanistic challenges: technology alienation, doctor-patient communication, teamwork, clinical thinking

Over-reliance on AI and VR technology may make residents simplify complex medical behaviors to pure technical operations, thus ignoring the emotional needs, social background and psychological state of patients, leading to the alienation tendency of “technology first”. The core of this tendency lies in the alienation of technological tools from auxiliary means to the center of learning and cognition, and the weakening of the patient-centered principle. For example, in VR simulation training, trainees often only need to focus on the identification of anatomical structures or the steps of surgical operations, without dealing with the fear, pain of patients or the anxiety of family members (79). This kind of de-contextualization training model, although effective in improving technical proficiency, may impair the ability of physicians to empathic.

The current mainstream AI and VR systems mostly focus on the training of individual skills, but seriously lack the simulation of “soft skills” such as doctor-patient communication, interdisciplinary collaboration, and leadership. However, the cultivation of these abilities is exactly one of the core contents of residency training. For example, in most VR scenarios, students cannot practice how to interpret medical conditions, obtain informed consent, or deal with doctor-patient conflicts (80).

Although team collaboration simulation (such as team exercise in the operating room) has begun to appear, its popularity rate is very low due to the high technical cost and complex scene design (81). This results in residents having to readjust to team work or feeling overwhelmed when faced with complex communication situations in the real clinical environment.

The virtual training environment is usually highly structured and cannot fully simulate the real clinical environment, so it is not possible to accumulate clinical experience and cultivate clinical acuity through various clinical uncertainties, and then form clinical thinking patterns.

## 5 Future perspectives

The future direction of development should not blindly pursue technology replacement, but should focus on the organic connection between technology iteration and real clinical practice. Firstly, it is necessary to promote the breakthrough of core technologies such as fine anatomy, tactile feedback, dynamic physiological factor loading, so that the virtual environment can more realistically simulate the high-risk scenarios such as anatomical variation, intraoperative bleeding, and nerve injury, and improve the authenticity and reliability of simulation. Secondly, a multi-dimensional standardized evaluation system should be established to correlate the operation data (such as instrument trajectory and strength curve) in virtual training with the actual clinical performance and combine it with the tutor’s evaluation to verify the effectiveness of skill transfer. Thirdly, the integration of medical education should be carried out in stages and steps: AI-VR is used for basic skills training in the early stage, the transition to high-fidelity simulator to deal with complex cases in the middle stage, and the return to clinical practice with real-time feedback from teachers in the late stage. In the future, through increasingly complex simulations, enhanced physiological modeling, personalized learning pathways with expanded AI capabilities, and better integration of technical and non-technical skill development, the balance between technical efficiency and the essence of serving the clinic and serving the education will be achieved (14).

The key to future development is not to pursue a complete replacement of the traditional model by technology, but to build complementary strategies. Resident training should adhere to the fundamental principle of “people-oriented, technology-based”. Technology should be viewed as a highly effective adjunct, not a definitive replacement for clinical practice. Only by finding a balance between technological empowerment and humanistic return can we avoid falling into the trap of technological determinism and truly realize the leap of medical education from “skill training” to “comprehensive training”. This is not only the direction of technical development, but also the inevitable requirement for the return of the essence of medical education. Finally, it is necessary to establish a strict quality certification system and ethical review mechanism, clarify the legal boundary and responsibility of technology application, and truly realize the rule of law and the rule of man. With these advances, the eventual realization is to train competent and compassionate physicians who can provide quality care to patients (82).

## 6 Limitations of this review

This review has several limitations. First, it is a scoping review rather than a formal systematic review, so meta-analytic pooling was not performed, and findings are descriptive rather than quantitative. Second, included studies vary widely in design, sample size, and outcome measures, limiting direct comparability. Third, most evidence comes from single-center, short-term evaluations; long-term retention of skills and real clinical outcomes (e.g., complication rates) remain understudied. Fourth, cost-effectiveness and equity of access data are sparse, especially in low-resource settings. Fifth, while we searched major databases, gray literature and unpublished abstracts were not included, potentially introducing publication bias. Finally, we did not conduct formal risk-of-bias assessment for all included studies, though we prioritized higher-quality designs (RCTs, validated assessments) in synthesis.

## 7 Discussion and conclusion

As cutting-edge tools in the field of medical education, AI and VR technologies have been widely promoted and applied to residency training in recent years, covering intelligent assisted diagnosis, surgical simulation, clinical skills training and other links, which are beneficial supplements and assistants to traditional teaching methods. The application of these techniques in the teaching of thyroid surgery has extended from preoperative diagnosis to surgical scenarios, and it is expected to systematically improve the anatomical cognition, operation proficiency and clinical decision-making ability of residents, thereby shortening the learning curve and improving the training efficiency (83).

AI technology has shown practical value in improving the thyroid ultrasound scanning skills and imaging diagnosis ability of residents. The intelligent ultrasound training system combines a sequential AI model with a deep learning architecture to provide objective, real-time and refined evaluation of the probe operation, scan trajectory, and image acquisition process, which is an advantage that cannot be achieved by traditional subjective tutor guidance. The system can distinguish the operation level of experts and novices with high accuracy, and provide quantitative feedback to help students improve their skills quickly, while unifying the evaluation criteria of different trainees (17). In addition to basic operational skills, AI-assisted diagnostic tools can analyze thyroid ultrasound images, assess the malignant risk of nodules and generate structured reports with the help of TI-RADS grading system, showing comparable diagnostic efficacy to experts, providing an objective and quantitative reference tool for residents to learn ultrasound interpretation (84). The adaptive learning platform can identify the shortcomings of trainees’ personalized knowledge (such as the difficulties in the interpretation of follicular lesions) and push targeted learning content to meet the multidisciplinary characteristics of thyroid disease diagnosis and treatment.

VR simulation platform and AI tools are complementary to each other, aiming to break through the limitations of traditional teaching such as lack of risk-free and repeatable and difficult practice opportunities (such as thyroidectomy surgery and fine needle aspiration biopsy) and lack of evaluation criteria (85). Modern VR simulator integrates high-fidelity 3D anatomical model and haptic feedback technology, which can restore real tissue resistance and surgical details such as nerve separation and parathyroid protection, and can provide a safe, repeatable and standardized learning environment (56). It has shown significant potential in improving the technical skills, hand-eye coordination and reducing errors of residents (17). Studies have shown that with repeated practice in the immersive environment, residents completed simulated thyroid surgery faster, had higher objective scores, and were significantly more satisfied with their training than those in the traditional teaching group. When VR is combined with AI analysis technology, the system can capture refined data such as instrument movement efficiency, hand stability, and error rate, generate objective and long-term skill assessment reports, and provide guidance for targeted training. In summary, AI and VR technology have brought unprecedented opportunities for residency training, which have shown significant advantages in skill training standardization, operation safety improvement and high-risk scenario simulation.

The application of AI and VR technology in the resident training is the enrichment of the form of medical education and the innovation of the concept. However, the current application of technology is far from mature. The application of these techniques in thyroid surgery, a specific and fine surgical subspecialty, also faces some obstacles and challenges, exposing some disadvantages and limitations. From the perspective of ethics, fairness and security, algorithm bias may lead to unfair training evaluation, and may pose serious challenges to data privacy and security when collecting and processing a large number of patient and trainee data (86). There are still technical defects, and there are doubts about the decision-making transparency and generalization ability of AI algorithm in complex and atypical cases (86). The fidelity of VR simulation, the accuracy of haptic feedback, and the differences in stress, teamwork and accident handling between VR simulation and real clinical environment may affect the transfer of training effects to real clinical scenes (87). In terms of matching balance, over-reliance on technological tools may weaken the tacit knowledge transmission and clinical thinking shaping in the original mentorship inheritance, and standardized AI feedback is difficult to completely replace the personalized and contextualized guidance provided by senior clinical tutors based on rich experience (88). Economically, the high basic cost, software customization development and continuous maintenance and upgrading cost aggravates the imbalance of medical education resource allocation (88). In terms of humanities, technological intervention may reduce the opportunities for direct interaction between doctors and patients, teachers and students, and virtual environments cannot fully reproduce the humanistic complexity in real medical situations (85). Therefore, in-depth review of these multi-dimensional limitations is essential for guiding the integration of technological rationality into medical education and ensuring the quality of resident training.

Future development must adhere to the balanced concept of “human-centered, technology-assisted,” clarifying that technology serves as a very effective supplement to traditional clinical mentor guidance, rather than a replacement. The core development directions include: breaking through key technological bottlenecks to improve the precision of anatomical simulation, acquire the ability of dynamic physiological simulation, and enhance the realism of haptic feedback, so that the simulation scenarios can be infinitely close to the real surgery procedures; establishing a multi-dimensional standardized evaluation system to correlate virtual training data with actual clinical assessments, thereby verifying the value of skill transfer; rationally allocating resources, optimizing learning paths, and facilitating their phased integration into residency training curricula. At the same time, it is essential to improve the governance system: formulating strict data privacy protection standards, mechanisms to avoid algorithmic bias, and quality certification standards for VR and AI tools, ensuring ethical compliance, fairness, accessibility, and efficiency in technology application. In the process of integrating AI and VR technologies into traditional residency training, we should also pay attention to the cultivation of doctor-patient communication and teamwork skills, which cannot obtained through new technologies. In the end, the ultimate goal of technological integration is not to replace human educators, but to empower them, thereby cultivating thyroid specialists who combine solid diagnostic and therapeutic abilities with humanistic care. While adhering to the essence of medicine, they will provide high-quality medical services to patients.
